# A Robust Quasi-Quantum Walks-based Steganography Protocol for Secure Transmission of Images on Cloud-based E-healthcare Platforms

**DOI:** 10.3390/s20113108

**Published:** 2020-05-31

**Authors:** Bassem Abd-El-Atty, Abdullah M. Iliyasu, Haya Alaskar, Ahmed A. Abd El-Latif

**Affiliations:** 1 Centre of Excellence in Cybersecurity, Quantum Information Processing, and Artificial Intelligence, Menoufia University, Shebin El-Koom 32511, Egypt; bassimeldeeb@gmail.com; 2 Electrical Engineering Department, College of Engineering, Prince Sattam Bin Abdulaziz University, Al-Kharj 11942, Saudi Arabia; 3 School of Computing, Tokyo Institute of Technology, Yokohama 226-8502, Japan; 4 School of Computer Science and Technology, Changchun University of Science and Technology, Changchun 130022, China; 5 Computer Science Department, Prince Sattam Bin Abdulaziz University, Al-Kharj 11942, Saudi Arabia; haya2alaskar@gmail.com; 6 Mathematics and Computer Science Department, Faculty of Science, Menoufia University, P.O. Box 32511, Shebin El-Koom 32511, Egypt; 7 School of Information Technology and Computer Science, Nile University, 26th July Corridor, Sheikh Zayed City, Giza 12588, Egypt

**Keywords:** quantum walks, steganography, medical image, cloud computing, E-healthcare, health informatics

## Abstract

Traditionally, tamper-proof steganography involves using efficient protocols to encrypt the stego cover image and/or hidden message prior to embedding it into the carrier object. However, as the inevitable transition to the quantum computing paradigm beckons, its immense computing power will be exploited to violate even the best non-quantum, i.e., classical, stego protocol. On its part, quantum walks can be tailored to utilise their astounding ‘quantumness’ to propagate nonlinear chaotic behaviours as well as its sufficient sensitivity to alterations in primary key parameters both important properties for efficient information security. Our study explores using a classical (i.e., quantum-inspired) rendition of the controlled alternate quantum walks (i.e., CAQWs) model to fabricate a robust image steganography protocol for cloud-based E-healthcare platforms by locating content that overlays the secret (or hidden) bits. The design employed in our technique precludes the need for pre and/or post encryption of the carrier and secret images. Furthermore, our design simplifies the process to extract the confidential (hidden) information since only the stego image and primary states to run the CAQWs are required. We validate our proposed protocol on a dataset of medical images, which exhibited remarkable outcomes in terms of their security, good visual quality, high resistance to data loss attacks, high embedding capacity, etc., making the proposed scheme a veritable strategy for efficient medical image steganography.

## 1. Introduction

In tandem with the advancements and accelerated growth of biomedical systems, medical data have assumed more sophisticated and ubiquitous roles in the modern age. Given secure communication channels, medical data can be securely stored in cloud-based E-healthcare platforms for wide ranging applications and uses related to education, research, medical consultations, etc. [1]. Due to concerns pertaining to patient confidentiality, today, data privacy and security are paramount when transferring medical information across the internet and storing it on cloud data storage facilities [2]. Medical data can be secured via appropriate cryptographic or data hiding techniques prior to uploading to the cloud. In cryptography, confidential data is obfuscated from an intelligible form to an incomprehensible pattern [3,4]. Meanwhile, in traditional data hiding, confidential information is imperceptibly suffused into some host media. Depending on its objective, data hiding can be further divided into watermarking for copyright protection, and steganography for securing confidentiality [5,6]. To elaborate, in steganography, a secret image is hidden in a host (or carrier) image making it useful in applications to restrict access to such confidential information [7]. In image steganography, the goal is to suffuse the confidential data into the spatial or transformation domain of the host (or carrier) image via carefully crafted alterations that retain the visual and statistical features of the host image [8].

Recently, many image steganography schemes that exploit the spatial domain have been studied extensively [8,9,10,11,12,13,14,15]. These spatial domain image steganography strategies involve direct substitution of some bits in pixel values of the cover image with secret bits [16]. The easiest type of this mechanism is the Least Significant Bit (LSB) technique where the confidential data is hidden in the LSBs of pixel values in the carrier image. The advantage of this approach is its high embedding capacity, while its main downside is its vulnerability to various forms of attacks. The common method to avoid this shortcoming is to encrypt the confidential data prior to hiding it into the cover image.

Usually, this shortcoming is mitigated by encrypting a well-designed image steganography mechanism that can withstand extraction of the hidden object from the cover image. Therefore, the security of such image steganography mechanisms can be enhanced by combining the steganography mechanism with a well-designed cryptographic mechanism, for instance, encrypting the secret object prior embedding it into the cover image or encrypting the stego image.

Meanwhile, notwithstanding these improvements, the inevitable realisation of scalable quantum computing resources portends huge implications to the security and utility of many information security paradigms. This is attributed to their stupendous computing power [17,18,19] of this new computing model, which means ordinarily intractable computations can be completed within minutes. This potentially implies loss of confidentiality and security in many of today’s data privacy applications, such as credit card transactions, etc. [19]. Considering this and in order to guarantee security against unauthorised breaches, it is important to consider infusing some level of quantumness into prevailing protocols. Despite the renewed impetus, funding, and progress in the road towards realisation of physical quantum computing hardware, such scalable technology is still unavailable [19]. Notwithstanding, this study explores the integration of quantum-inspired controlled alternate quantum walks (CAQWs) into medical image steganography. In doing so, we build on the available literature with both quantum image processing (QIP) [19,20] focus and other quantum-inspired approaches like our own [21]. For example, in [12], El-Latif et al. proposed an image steganography mechanism based on quantum substitution boxes whose construction is based on one-dimensional two-particle quantum walks (1D 2P QWs). Similarly, in [13], Peng et al. suggested a new image steganography mechanism based on 1D 2P QWs to embed a greyscale image into a colour image, where 1D 2P QWs were used to decide which colour channel will host the secret bits. Meanwhile, motivated by the conclusion, in [22], which inferred that the realisation of CAQWs is easier than that of 1D 2P QWs, in this study, we tailor our embedding and extraction processes in tandem with that inference. Specifically, quantum-inspired variants of CAQWs are used to determine pixels for overlaying secret (or hidden) bits in the carrier image. Our design precludes the need for pre or post encryption and extraction procedures which means that only stego image and primary states of the CAQWs are required to extract the hidden image. For further illustration, Table 1 provides a comparative analysis between our proposed mechanism relative to other data hiding mechanisms that are built on quantum mechanics. Whereas these data hiding mechanisms require the availability of quantum computing hardware (i.e., to implement them in real time), execution of our proposed schemes is confined to digital computers. Simulation-based experiments validate the efficacy of our proposed quantum-inspired protocol as exhibited via outcomes that offer good visual quality, resistance to data loss attacks, high embedding capacity, and robust security.

The remainder of our study flows as follows: background on the framework for secure transmission in cloud-based E-healthcare together with a brief overview of quantum walks are presented in Section 2. That background provides foundation for our proposed image steganography scheme and its composition as presented in Section 3. Finally, the experimental implementation of the proposed framework and discussions of results therefrom are presented in Section 4.

## 2. IoT-Based Healthcare and Quantum Walks

### 2.1. Framework for Secure Transmission of Images on Cloud-Based E-Healthcare Platforms

The Internet of Things (IoT) offers highly scalable computing resources as online services. With the rapid development of cloud computing technologies, an expanding number of people, institutions and companies are choosing cloud platforms to store and manipulate their data. Cloud computing has enormous advantages that include remote storage, mobility, data sharing, cost savings in hardware and software, etc. However, many security challenges that are attributed to the cloud computing environment have not yet been addressed, especially in traditional computing environments [24,25]. Further, it has been observed that security and privacy issues have severely restricted the practical applications of cloud technologies [26]. To address these significant problems, it is essential to propose and design new algorithms and methods to secure cloud computing infrastructure.

**Figure 1:**
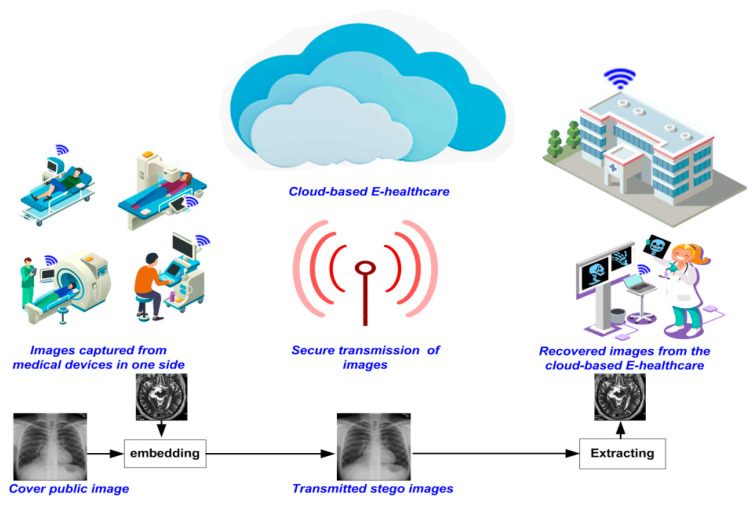
General framework envisioned for secure image transmission on cloud-based E-healthcare platforms.

Meanwhile, advances in quantum technologies are expected to usher in the quantum era whence there will be adequate computing power to tamper with the best of today’s traditional cryptographic algorithms [27,28]. Already, numerous quantum algorithms have been validated at small scale and lab level as well as via simulations. Presently, there is immense interest in the area, which is supported by extensive investments by governments, industry, and academia. [19]. Indeed, this race for quantum supremacy supports the consensus that the inevitable quantum era is upon us. In the meantime, to forestall future violations of today’s data security, their protection using secure pseudo-quantum or quantum-inspired algorithms seems a worthwhile undertaking. Our proposed framework for the safe transfer of medical images on cloud-based E-healthcare platforms is outlined in Figure 1. From this figure, we see that secret information is infused onto the medical images captured from patients prior to uploading them as part of a public dataset. Elsewhere in the system (Figure 1), authorised users, such as doctors, healthcare providers, etc., download the stego image (which carries the secret medical information) and then extract the secret information using our pseudo-quantum walks mechanism. While this secret information could be any type of media such as patient records, identification, etc., in this study, different types of medical images are used. The implementation of our quantum-inspired quantum walks scheme provides cogency supporting seamless refinements to safeguard the images from probable attacks when quantum computing hardware inevitably become available [19].

### 2.2. Quantum Walks

Controlled alternate quantum walks (CAQWs) have two essential elements: the first one is the walker space $H_{p}$ and the other one is the coin space $H_{c}=\cos\theta |0\rangle +\sin\theta |1\rangle$ both of which reside within a Hilbert space $H=H_{p}⊗H_{c}$ [4]. The coin particle is a quantum system existing in a two-dimensional Hilbert space with amplitudes cosθ and sinθ. In every step of executing a *B*-bit string CAQWs on a *V* node circle, an evolution operator ${\hat{T}}_{0}$ (or ${\hat{T}}_{1}$) is applied when the ith bit of bit string *B* is 0 (or 1). The unitary transformations ${\hat{T}}_{0}$ and ${\hat{T}}_{1}$ are defined in Equation (1).

(1) $$\begin{array}{l} {\hat{T}}_{0}={\hat{F}}_{y}\left(\hat{I}⊗{\hat{R}}_{0}\right){\hat{F}}_{x}\left(\hat{I}⊗{\hat{R}}_{0}\right)\\ {\hat{T}}_{1}={\hat{F}}_{y}\left(\hat{I}⊗{\hat{R}}_{1}\right){\hat{F}}_{x}\left(\hat{I}⊗{\hat{R}}_{1}\right) \end{array}$$

where ${\hat{F}}_{x}$ denotes to the shift operator of CAQWs on a ring with *V* nodes acting on *x* dimensions in the form defined in Equation (2).

(2) $${\hat{F}}_{x}=\sum_{x,y}^{v}\left(\left|\left(x+1\right)\mathrm{mod}V,y,0\rangle \langle x,y,0\right|\right)+\sum_{x,y}^{v}\left(\left|\left(x-1\right)\mathrm{mod}V,y,1\rangle \langle x,y,1\right|\right)$$

Like ${\hat{F}}_{x}$, ${\hat{F}}_{y}$ expresses CAQWs on a ring with *V* nodes acting on *y* dimensions, while the two operators ${\hat{R}}_{0}$ and ${\hat{R}}_{1}$ are coin operators defined as in Equation (3).

(3) $$\begin{array}{l} {\hat{R}}_{0}=\left(\begin{array}{ll} \cos\;\alpha_{0} & \sin\;\alpha_{0}\\ \sin\;\alpha_{0} & -\cos\;\alpha_{0} \end{array}\right)\\ {\hat{R}}_{1}=\left(\begin{array}{ll} \cos\;\alpha_{1} & -\cos\;\alpha_{1}\\ \sin\;\alpha_{1} & \sin\;\alpha_{1} \end{array}\right) \end{array}$$

After *i* steps, the final state of $|Q\rangle_{\mathrm{initial}}$ can be expressed in the form presented in Equation (4).

(4) $$\left|Q\rangle_{i}={\left(\hat{T}\right)}^{i}\right|Q\rangle_{initial}$$

Eventually, the possibility of detecting the walker of CAQWs at location (x, y) after *i* steps can be computed using Equation (5).

(5) $$P\left(x,y,i\right)=\sum_{s\in \{0,1\}}\left|\langle x,y,s\right|{\left(\hat{T}\right)}^{i}|Q\rangle_{0}{|}^{2}$$

These mathematical formulations are modelled as building blocks of our quantum-inspired scheme and deployed to safeguard medical images in an e-healthcare scenario. Details of our rendition of the quantum walks protocol on digital computers is presented in the next section.

## 3. Proposed Image Steganography Scheme

**Figure 2:**
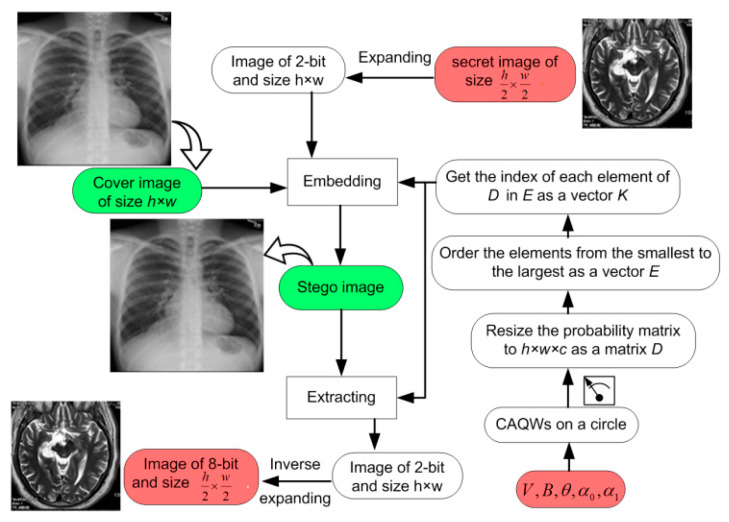
Outline of the proposed image stego scheme.

In this section, we illuminate the role of CAQWs in designing an image steganography mechanism to embed a confidential image onto a carrier image. The main role of CAQWs is to determine two LSB pixel locations in the carrier image to utilise for embedding the secret bits. However, only two bits of the secret image are embedded into the two LSBs of the selected pixel. Therefore, the confidential and carrier images are $\frac{h}{2}\times \frac{w}{2}$ and *h × w* in dimension, whilst the outcome capacity is 2 bits per pixel. Figure 2 presents the general framework of our proposed quantum-inspired image steganography mechanism, while execution of the embedding process is outlined in Algorithm 1. 

First, a vectorisation procedure uses CAQWs to transform the detection matrix in (5) as well as key information about the carrier and confidential images into a vector that is subsequently used as part of the embedding process. The initial key parameters ($V,\;B,\;\theta ,\;\alpha_{0},\;\alpha_{2}$) are utilised to execute CAQWs on a cycle of *V* odd-valued nodes determined by a bit string *B* to retrieve a *V × V* probability distribution matrix, *P*. Here, the primary state of the coin walker is $H_{c}=\cos\theta |0\rangle +\sin\theta |1\rangle$ wherein $0\leq \theta ,{\;\alpha }_{0},{\;\alpha }_{2}\geq \pi /2$ are the key parameters used to construct the coin operators ${\hat{R}}_{0}$ and ${\hat{R}}_{1}$, respectively.
**Algorithm 1:** Embedding process**Input:** Carrier image (*CIm*), Confidential image (*SIm*), and Initial key parameters ($V,\;B,\;\theta ,{\;\alpha }_{0},{\;\alpha }_{2}$)**Output:** Stego image (Stgo)*P*← CAQWs ($V,\;B,\;\theta ,{\;\alpha }_{0},{\;\alpha }_{2}$) // Operate CAQWs using initial key parameters[*h*, w, c] ← size (*CIm*) // Obtain the size of the carrier image*D*← resize (*P*, [h, w × c ]) // Resize the matrix P to the dimension of the cover image*E*← order (*D*) // Order the elements of *D* in decreasing order*K*= index (*D*, *E*) // Obtain the index of each element of *D* in *E**ESIm* ← expand (*SIm*) // Expand the 8-bit and $\frac{h}{2}\times \frac{w}{2}$ dimensional confidential image *SIm* to a 2-bit image of *h × w* dimension.// Transform the expanded secret image *ESIm* and the carrier image *CIm* into vectors*SVec*← reshape (*ESIm*, 1, h × w × c)*CVec*← reshape (*CIm*, 1, h × w × c)**//** Embedding processfor i←1 to h × w × c   *StgoVec*(k(i))←Replace 2LSBs of *CVec*(k(i)) with 2bits of *SVec*(i);end// Transform the vector *StgoVec* into an imageStgo←reshape (*StgoVec*, h, w, c)

## 4. Simulation-Based Experiments

**Figure 3:**
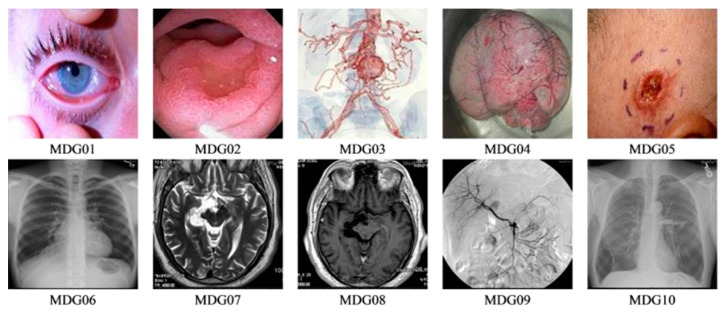
Dataset showing carrier and secret images used in the experiments.

To appraise the performance of our proposed image steganography mechanism, various analyses are presented to assess stego image quality, data loss analysis, security analysis, and payload capacity. We used a workstation equipped with a laptop with Intel ${\mathrm{core}}^{\mathrm{TM}}$ i5, a 6-GB RAM and a preinstalled MATLAB R2016b software. Our dataset comprises of two sets of medical images sourced from the MedPix dataset in [29]. The first, labelled as MDG01 through MDG05, consists of two sets of five 256 × 256 and 128 × 128 colour images, while the second set contains two sets of five greyscale medical images (labelled MDG06 through MDG10) of the same dimensions, i.e., 256 × 256 and 128 × 128. We reiterate that, as noted earlier, while the cover information can be public or private different media (text, images, patient records, etc.), in keeping with the scope of our study, we choose to implement our scheme using medical images that can be deployed in E-healthcare platforms. Therefore, the 256 × 256 images of both sub-datasets will be used as cover images whereas the 128 × 128 images constitute the secret (or confidential) images to be used in validating our proposed scheme. Although each sub-dataset consists of ten cover and carrier images, altogether 10 image types (presented in Figure 3) are used in the reported experiments. Furthermore the initial key parameters for operating CAQWs on a circle are set as *V = 21, B*= “1011 0110 0010 0111 0101 0101 0101 1010 0”, θ=π/2, $\alpha_{0}=\pi /4$ and $\alpha_{1}=\pi /6$.

### 4.1. Image Quality Analysis

Numerous tests are employed in assessing the quality of stego images emanating from our proposed scheme. These metrics include the Peak Signal to Noise Ratio (PSNR), Structural Similarity Index Metric (SSIM), Universal Image Quality (UIQ), Normalised cross correlation (NCC), Normalised Absolute Error (NAE), Image Fidelity (IF), Average difference (AD), Maximum difference (MD), and Structural Content (SC) to appraise it performance in terms of image quality. As a prelude to our validation, definitions of the metrics used as well as overview of their established benchmarks are presented in the remainder of this subsection. 

#### 4.1.1. Peak Signal to Noise Ratio (PSNR)

**Figure 4:**
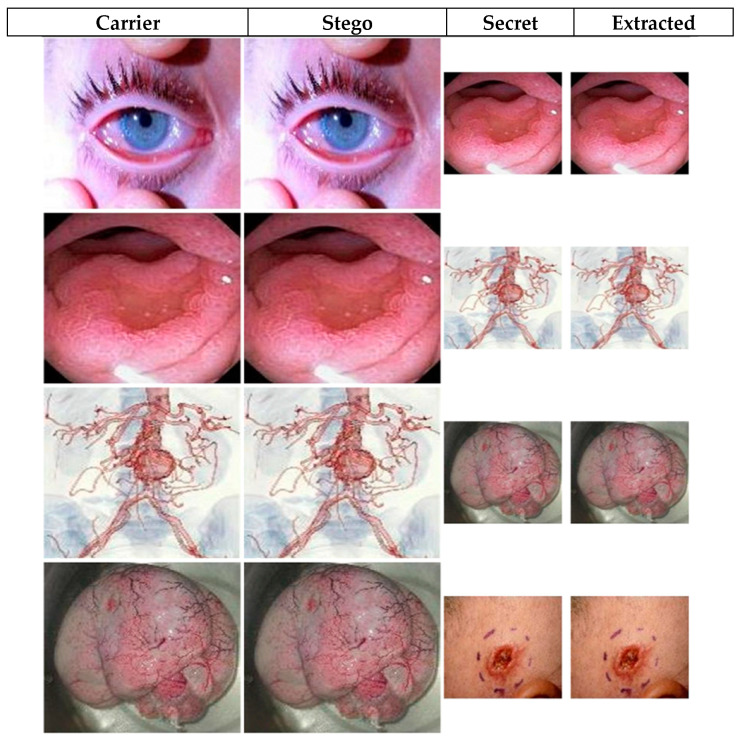
Visual quality for marked (i.e. stego) versions of colour images in Figure 3.

**Figure 5:**
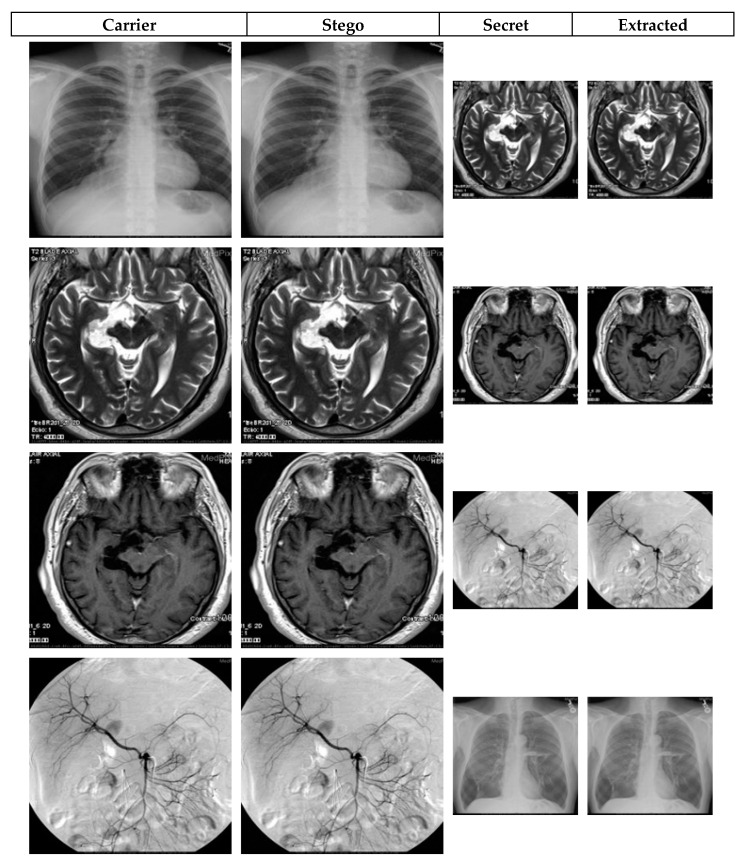
Visual quality for marked (i.e. stego) versions of greyscale images in Figure 3.

**Figure 6:**
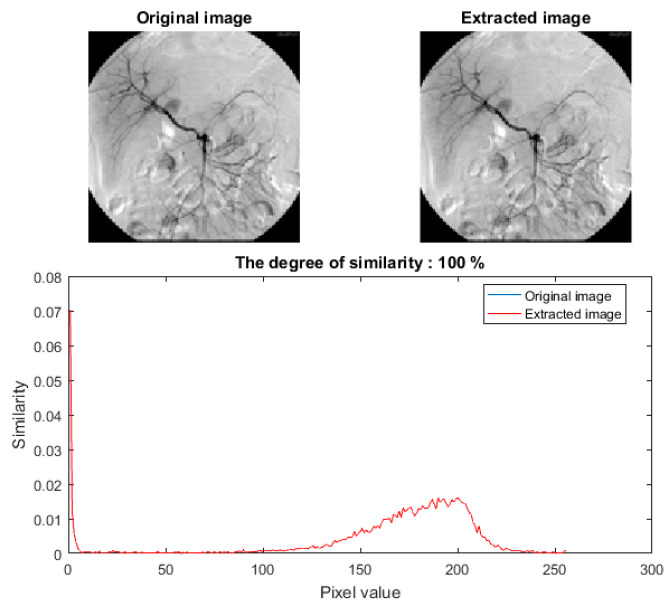
Histogram showing similarity of the secret image MDG09 prior to and after its extraction from the stego image MDG06.

PSNR is a test to measure stego image quality. It can be expressed mathematically as:

(6) $$\begin{array}{l} PSNR\left(C,S\right) \end{array}\begin{array}{l} 20\log_{10} \end{array}\begin{array}{l} \left(\frac{MAX_{C}\times \sqrt{h\times w}}{\sqrt{\sum_{i=0}^{h-1}\sum_{j=0}^{w-1}{\left[C\left(i,j\right)-S\left(i,j\right)\right]}^{2}}}\right) \end{array}$$

where *MAX*_c_ indicates the maximum pixel values of the carrier image *C*, and *S* indicates its corresponding stego image. Both the carrier and stego images are *h* × *w* pixels in dimension. The visual quality of stego images reported in Figure 4 and Figure 5 attests to the high performance, i.e., the imperceptibility, of our technique. The figures show each original image and its stego version as well as the secret image prior to and after extraction from the stego image. Further, a plot of the variation of a sample of histogram similarity [30] for the secret image prior to and after extraction from the stego image presented in Figure 6. Similarly, the PSNR values reported in Table 2 and Table 3 show the quantitative assessment of fidelity between the original and stego versions of the carrier and secret images. High PSNR values indicate good quality, where the naked eye cannot detect the dissimilarity between the stego image and its cover version.

#### 4.1.2. Structural Similarity Index Metric (SSIM)

SSIM is a metric used to distinguish pristine (C) and stego (S) versions of an image. It is defined in the form presented in Equation (7).

(7) $$SSIM\left(C,S\right)=\frac{\left(2\mu_{C}\mu_{S}+C_{1}\right)\left(2\sigma_{C,S}+C_{2}\right)}{\left(\mu_{C}^{2}+\mu_{S}^{2}+C_{1}\right)\left(\sigma_{C}^{2}+\sigma_{S}^{2}+C_{2}\right)}$$

where, *C*_1_ and *C*_2_ are constants, *S* and μ are the variance and mean, respectively. A typical SSIM value varies in the range [0, 1], where values closer to 1 indicate better match between the pristine and altered pairings of the carrier and stego images. The values of SSIM for our scheme are recorded in Table 2 for pairings from our first sub-dataset comprising of colour images and in Table 3 for the sub-dataset of greyscale medical images.

#### 4.1.3. Universal Image Quality (UIQ)

UIQ is another metric used to distinguish pristine (C) and stego (S) that is defined mathematically as:

(8) $$UIQ\left(C,S\right)=\frac{4\sigma_{C,S}\mu_{C}\mu_{S}}{\left(\mu_{C}^{2}+\mu_{S}^{2}\right)\left(\sigma_{C}^{2}+\sigma_{S}^{2}\right)}$$

where, *s* and *m* are the variance and mean, respectively. The UIQ value takes the range [−1, 1] and values in Table 2 and Table 3 report UIQ values obtained from our experiments with the colour and greyscale medical image sub-datasets, respectively. In both tables, we note the reported UIQ values are close to 1 for both sub-datasets.

#### 4.1.4. Normalised Cross Correlation (NCC)

NCC is an effective metric that is used to distinguish pristine (C) and stego (S) images. It is computed using the formulation in Equation (9).

(9) $$\begin{array}{l} NCC\left(C,S\right)=\frac{\sum_{i=1}^{h}\sum_{j=1}^{w}\left(C\left(i,j\right)-\mu_{C}\right)\left(S\left(i,j\right)-\mu_{S}\right)}{\sqrt{\sum_{i=1}^{h}\sum_{j=1}^{w}{\left(C\left(i,j\right)-\mu_{C}\right)}^{2}}\sqrt{\sum_{i=1}^{h}\sum_{j=1}^{w}{\left(S\left(i,j\right)-\mu_{S}\right)}^{2}}} \end{array}$$

where *m*_c_ and *m*_s_ are the means of the carrier and stego images, respectively. Like UIQ, the NCC values take the range [−1, 1], where values closer to 1 indicate better match between the pristine and altered pairings of the carriers and stego images [30]. As reported in Table 2 and Table 3, NCC values obtained from our scheme exhibit fidelity between the cover and altered images used in both sub-datasets of our experiments since all the reported values are close to 1.

#### 4.1.5. Normalised Absolute Error (NAE)

Another effective metric used to distinguish pristine (C) and stego (S) images is NAE which is formulated as presented in Equation (10).

(10) $$NAE\left(C,S\right)=\frac{\sum_{i=1}^{h}\sum_{j=1}^{w}\left(C\left(i,j\right)-S\left(i,j\right)\right)}{\sum_{i=1}^{h}\sum_{j=1}^{w}\left(C\left(i,j\right)\right)}$$

NAE values closer to 0 indicate concordance between the pristine and altered pairings of the carrier and stego images. For our proposed mechanism, these values are presented in Table 2 and Table 3 respectively for the colour and greyscale medical images reported in our experiments (i.e., Figure 3).

#### 4.1.6. Image Fidelity (IF)

Image fidelity (IF) is a metric used to distinguish pristine (C) and stego (S) images. Mathematically, IF is computed using the formulation in Equation (11).

(11) $$IF\left(C,S\right)=1-\frac{\sum_{i=1}^{h}\sum_{j=1}^{w}{\left(C\left(i,j\right)-S\left(i,j\right)\right)}^{2}}{\sum_{i=1}^{h}\sum_{j=1}^{w}{\left(C\left(i,j\right)\right)}^{2}}$$

Generally, IF values closer to 1 are desirable as an indicator of fidelity between pairings of pristine and altered images. For our two sub-datasets, IF results are reported in Table 2 and Table 3 for the colour and greyscale images, respectively.

#### 4.1.7. Average Difference (AD)

AD is another metric used to measure the average difference between pristine (C) and stego (S) versions of an image. It is expressed in the format presented in Equation (12).

(12) $$AD\left(C,S\right)=\frac{\sum_{i=1}^{h}\sum_{j=1}^{w}\left(C\left(i,j\right)-S\left(i,j\right)\right)}{h\times w}$$

AD values closer to 0 indicate better fidelity between the pristine and altered pairings of the carrier and stego images. For the colour and greyscale images used in our experiment, AD values are presented in Table 2 and Table 3, respectively. Both tables report outcomes close to 0 for the pairings indicated.

#### 4.1.8. Maximum Difference (MD)

MD is the maximum error signal between pristine (C) and stego (S) versions of an image. Mathematically, MD is expressed as:

(13) MD(C,S)=MAX|C(i.j)−S(i,j)|

Table 2 and Table 3 present MD values for the colour and greyscale medical images reported in our experiment. It is noteworthy that all values reported are equal to 3, which is the established benchmark for the MD metric [30].

#### 4.1.9. Structural Content (SC)

SC is also used to distinguish pristine (C) and stego (S) images using the formula presented in Equation (14).

(14) $$SC\left(C,S\right)=\frac{\sum_{i=1}^{h}\sum_{j=1}^{w}{\left(S\left(i,j\right)\right)}^{2}}{\sum_{i=1}^{h}\sum_{j=1}^{w}{\left(C\left(i,j\right)\right)}^{2}}$$

The values of SC closer to 1 indicate better match between the pristine and altered pairings of the carrier and stego images. SC values for our mechanism are presented in Table 2 for various pairings of colour images and in Table 3 for various greyscale medical images in our second sub-dataset. Throughout, our results are very close to 1 which is the required of benchmark for efficient stego schemes [30].

### 4.2. Data Loss Analyses

During data transfer, media are easily corrupted or damaged by noise or data loss. Therefore, an important test of a well-designed data hiding protocol is its ability to withstand data loss attacks [31].

**Figure 7:**
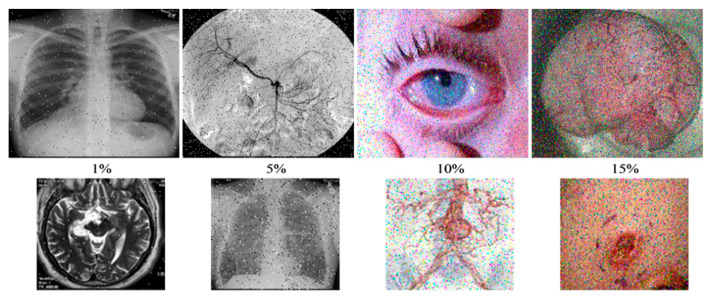
Stego and extracted images under noise of various densities. The first row shows the stego images with added Salt and Pepper noise and their corresponding extracted versions in the second row.

**Figure 8:**
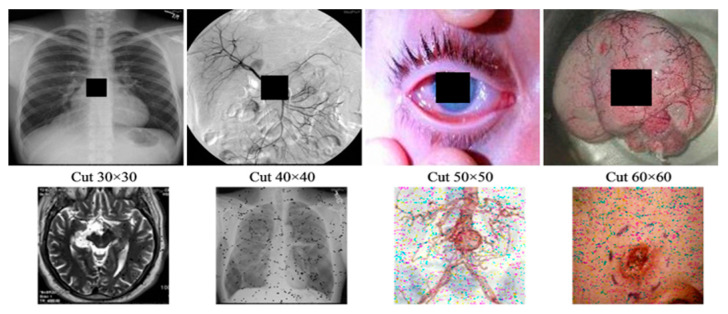
Stego and extracted images under various data loss by cutting different blocks. The first row shows the stego images with a block of data loss and their corresponding extracted versions in the second row.

To assess our proposed scheme in terms of different attacks, we considered clipping and noise addition tests. In the former, cut-out blocks were used to clip content of the stego image. In noise addition, we considered effects of adding "Salt and Pepper" noise to the stego images. Figure 7 and Figure 8 show the outcomes of the data loss (clipping) and noise addition attacks, respectively. From these outcomes, we note the ability to recover "useable", i.e., imperceptible, images despite the attacks mentioned. Moreover, since images from both attacks can be easily understood, it is apparent that the proposed scheme produces stego images capable of withstanding data loss and noise addition attacks.

### 4.3. Payload Capacity

The ability of a stego image to remain imperceptible is a function of its embedding capability. In this sense, payload capacity quantifies the proportion of data between the hidden and carrier bits. At 6 bits/24 bits (or 2 bits/8 bits), our proposed scheme offers adequate payload to accommodate large images whilst maintaining visual quality and withstanding different attacks.

### 4.4. Security Analysis

**Figure 9:**
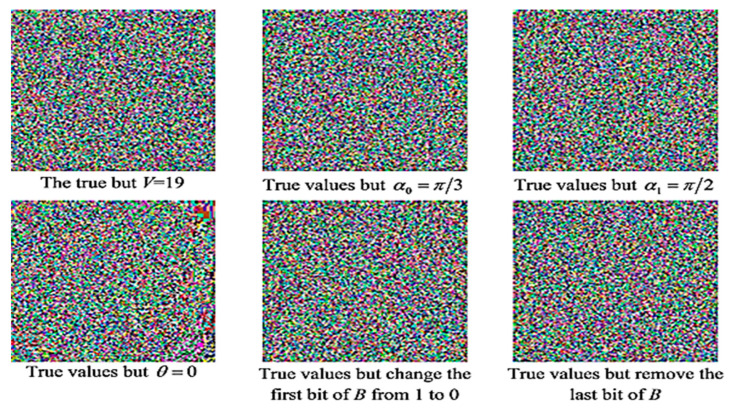
Extracted secret image MDG04 recovered from the stego image MDG01 for varying changes in key parameters.

The quantumness inherent to our pseudo-CAQWs provides its propriety against infringements expected when the inevitable transition to the quantum computing paradigm is made. Furthermore, the key space arising from the CAQW on a circle employed in our scheme, i.e., ($V,B,\theta ,\alpha_{0},\alpha_{1}$) provide adequate security against attempts to violate the security of stego images emanating from the proposed technique. Theoretically, the parameter *B* could extend to infinity, which implies an infinite length of a bit of string [30,31]. However, in mathematics and digital computing, precision can be measured by the number of decimal digits that are used to express it as a digital value [32]. Therefore, assuming a precision calculation of 10^−16^, then the key space for each key parameter is 10^16^, and therefore the key space for all parameters in our proposed technique is 10^80^. Like any cryptographic mechanism, if the full key parameters to execute CAQWs are revealed, then anyone can accurately retrieve the probability distribution required to violate the scheme [31]. On the other hand, if part or most of the parameters are concealed, it becomes impracticable to estimate the probability distribution. In designing our proposed scheme, any misstep in recovering the vector *K* (i.e., Step 4 of Algorithm 1) renders any attempt to retrieve the encryption key futile, which guarantees the security of images secured via the proposed technique. To validate this cogitation, we executed the extraction procedure to retrieve the secret image MDG04 from the stego MDG01 image within minuscule changes to its key. The outcomes reported in Figure 9 are evidence that, despite attempts to violate the stego image using diminutive adjustments to its key space, the extracted image remains indecipherable, which is a testimony of the strength of our protocol to withstand alterations to its key space. 

### 4.5. Discussion

Inspired by the utility and tamper-proof security offered by quantum computing, we have presented a robust medical image steganography protocol based on a quantum-inspired quantum walks protocol with the aim of securing transmission of images on cloud-based E-healthcare platforms. Unlike standard QIP-based approaches, the proposed technique is built on classical renditions of quantum mechanics, which, subject to appropriate refinements, makes it tenable for securing the images against the misuses feared when physical quantum hardware is realised. Specifically, the scheme utilises the potency of our quasi or bare bones classical transcription of CAQWs for both the embedding and extraction processes while the application is tailored on medical images. The quasi CAQW protocol is used to determine areas of the carrier image that the secret bits are overlaid. Our proposed design precludes the need for pre or post encryption and extraction procedures which implies that only the stego image and primary states of the CAQWs are required to extract the hidden image. Our proposed approach is extensively tested on a dataset comprising of colour and greyscale medical images (see Figure 3).

**Figure 10:**
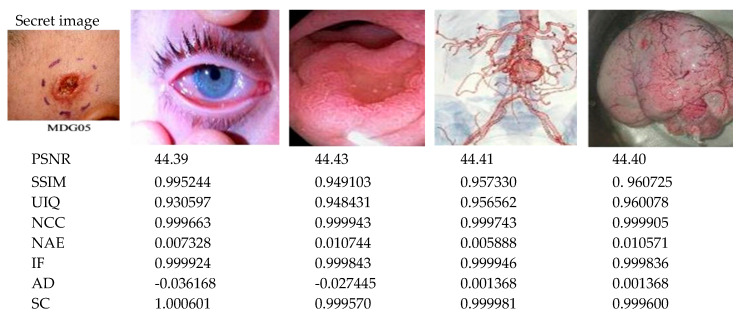
Performance analysis of the proposed protocol for colour medical images. Here, the secret medical image MDG05 is embedded onto all the remaining cover colour images (labelled MDG01 through MDG04 in Figure 3).

**Figure 11:**
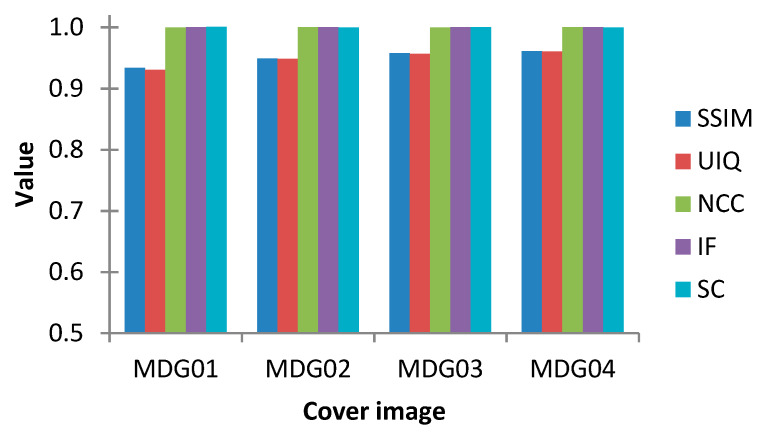
Graphical representation of performance analysis of the proposed steganography scheme where the secret medical image (MDG05) is embedded onto the remaining cover colour medical images presented earlier in Figure 3 (i.e., those labelled MDG01 through MDG04).

**Figure 12:**
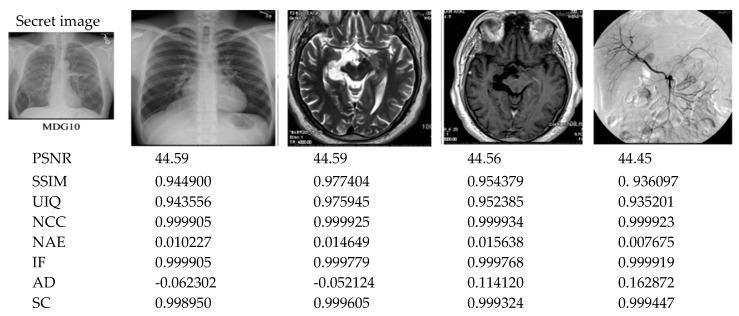
Performance analysis results of the proposed protocol. Here, the secret medical image MDG10 is embedded onto the remaining cover greyscale images shown (labelled MDG06 through MDG09 in Figure 3).

**Figure 13:**
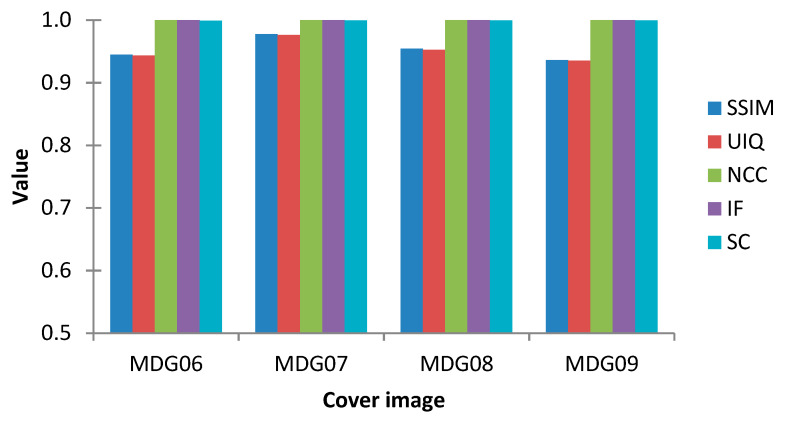
Graphical representation of performance analysis of the proposed steganography scheme where the secret medical image (MDG10) is embedded onto the remaining cover greyscale images presented earlier in Figure 3 (i.e., those labelled MDG06 through MDG09).

The twelve tests and measurements reported earlier in this section demonstrate the efficiency of our proposed protocol in terms of wide-ranging image and statistical metrics, including PSNR, SSIM, UIQ, NCC, NAE, IF, AD, and SC. Furthermore, Figure 10 presents outcomes and metrics obtained from embedding the secret medical MDG05 image (presented earlier in Figure 3) onto the cover colour images (labelled MDG01 through MDG04 in Figure 3). Additionally, Figure 11 presents the graphical representation of the SSIM, UIQ, NCC, IF and SC metrics for these pairings. Similarly, Figure 12 presents the outcomes from embedding the secret greyscale MDG10 image (presented earlier in Figure 3) onto the cover greyscale images shown (labelled MDG06 through MDG09 in Figure 3). Likewise, the graphical representation of these outcomes for the SSIM, UIQ, NCC, IF and SC metrics is presented in Figure 13. Despite the differences in their implementation (i.e., quantum-inspired and QIP-based [20]), in order to demonstrate the effectiveness of the proposed mechanism, Table 4 provides a comparative analysis of its performance relative to other simulation-based QIP data hiding methods in terms of embedding capacity as well as the mean values for PSNR, SSIM, and UIQ.

As reported in the table, our proposed technique fares relatively well alongside the schemes presented in [5,12,20].

## 5. Concluding Remarks 

Our study has presented a simple yet robust quasi-quantum walks-based image steganography mechanism to support secure transmission in cloud-based E-healthcare platforms. The embedding and extraction processes are tailored in terms of classical renditions of the controlled alternate quantum walks (i.e., CAQWs), which are subsequently used to determine pixels for overlaying secret (or hidden) bits in the stego image. The new steganography design precludes the need for pre or post encryption and extraction procedures, which means that only stego image and primary states of the CAQWs are required to extract hidden images. In addition, the new design simplifies the process of extracting confidential (i.e., hidden) information since only the stego image and primary states to execute the protocol are required. The proposed approach is tested on a dataset of colour and greyscale medical images using several simulation-based experiments. Outcomes validate the efficacy of the new scheme in terms good visual quality, resistance to data loss attacks, high embedding capacity, and robust security. Furthermore, the performance analysis shows that the proposed quantum-inspired scheme performs well relative to other state-of-art techniques, which suggests potential applications for the proposed scheme as a veritable strategy for efficient medical image steganography on future computing paradigms.

## Figures and Tables

**Table 1:** Comparative analysis between proposed protocol and similar quantum-based data hiding techniques.

Mechanism	Description	Capacity	Requirements for Extraction of Secret Image from Stego Image
Proposed	Designed as a quasi-quantum-inspired scheme where a secret colour (or greyscale) image is embedded onto a cover colour (or greyscale) image based on CAQWs. The role of CAQWs is used to determine the location of pixels in the carrier image to suffuse secret bits.	2-bit/8-bit	Control parameters for running CAQWs
Miyake et al. [5]	Simulation-based circuit model quantum image processing (QIP) [20] implementation of quantum greyscale image watermarking based on SWAP and CNOT gates.	2-bit/8-bit	Original carrier image and the key used in the scrambling process.
El-Latif et al. [12]	Simulation-based QIP protocol where a secret colour (or greyscale) image is embedded onto a cover colour (or greyscale) image based on quantum substitution boxes. The construction of quantum substitution boxes is based on 1D 2-Particle QWs, which requires more resources than CAQWs.	2-bit/8-bit	Quantum substitution boxes
Peng et al. [13]	Simulation-based implementation of 1D 2-Particle quantum walks to embed a secret greyscale image onto a cover colour image. 1D 2-Particle QWs is used to select one channel from (RGB) of the cover image to implant the secret bits.	2-bit/24-bit	Control parameters for running 1D 2-Particle QWs.
Li et al. [14]	A QIP protocol where a greyscale image is embedded onto a quantum colour image based on quantum Gray code.	2-bit/24-bit	Quantum Gray code
Zhou et al. [15]	A QIP scheme where greyscale images are embedded onto greyscale images based on Bit-plane, Swap gates, and Arnold image scrambling.	0.5-bit/8-bit	Original cover image and three types of keys (K, K1, and K2) each the size of the secret message.
El-Latif et al. [23]	A QIP data hiding mechanism suffusing greyscale images onto greyscale images based on the logistic chaotic map.	2-bit/8-bit	Control parameters for running the logistic map as well the key matrix generated from the embedding process whose size is the same size as the secret message.

**Table 2:** PSNR, SSIM, UIQ, NCC, NAE, IF, AD, MD, and SC values for colour images used in the experiments reported.

Measurement	Cover Image	Secret Image				
		MDG01	MDG02	MDG03	MDG04	MDG05
PSNR	MDG01	-	44.3009	44.0536	44.3985	44.3903
	MDG02	44.1068	-	44.0039	44.4310	44.4251
	MDG03	44.1238	44.3526	-	44.4266	44.4110
	MDG04	44.1346	44.3555	44.0179	-	44.4008
	MDG05	44.1059	44.3497	44.0078	44.4238	-
SSIM	MDG01	-	0.9331	0.9318	0.9338	0.9338
	MDG02	0.9470	-	0.9471	0.9491	0.9491
	MDG03	0.9556	0.9574	-	0.9572	0.9573
	MDG04	0.9594	0.9603	0.9593	-	0.9607
	MDG05	0.9674	0.9684	0.9675	0.9689	-
UIQ	MDG01	-	0.9299	0.9288	0.9306	0.9306
	MDG02	0.9463	-	0.9464	0.9484	0.9484
	MDG03	0.9548	0.9566	-	0.9564	0.9566
	MDG04	0.9588	0.9596	0.9587	-	0.9601
	MDG05	0.9673	0.9683	0.9673	0.9688	-
NCC	MDG01	-	0.9991	0.9999	0.9996	0.9997
	MDG02	0.9999	-	0.9999	0.9999	0.9999
	MDG03	0.9999	0.9992	-	0.9997	0.9997
	MDG04	0.9999	0.9991	0.9999	-	0.9999
	MDG05	0.9999	0.9993	0.9999	0.9998	-
NAE	MDG01	-	0.0074	0.0076	0.0073	0.0073
	MDG02	0.0111	-	0.0113	0.0107	0.0107
	MDG03	0.0061	0.0059	-	0.0059	0.0059
	MDG04	0.0109	0.0106	0.0110	-	0.0106
	MDG05	0.0099	0.0096	0.0100	0.0096	-
IF	MDG01	-	0.9999	0.9999	0.9999	0.9999
	MDG02	0.9998	-	0.9998	0.9998	0.9998
	MDG03	0.9999	0.9999	-	0.9999	0.9999
	MDG04	0.9998	0.9998	0.9998	-	0.9998
	MDG05	0.9998	0.9999	0.9998	0.9999	-
AD	MDG01	-	0.1220	−0.3018	0.0705	0.0362
	MDG02	−0.2475	-	−0.3654	0.0069	−0.0274
	MDG03	−0.2187	0.0872	-	0.0357	0.0014
	MDG04	−0.2315	0.0744	−0.3494	-	−0.0114
	MDG05	−0.2387	0.0672	−0.3566	0.0157	-
MD	MDG01	-	3	3	3	3
	MDG02	3	-	3	3	3
	MDG03	3	3	-	3	3
	MDG04	3	3	3	-	3
	MDG05	3	3	3	3	-
SC	MDG01	-	1.0015	0.9970	1.0009	1.0006
	MDG02	0.9965	-	0.9948	1.0001	0.9996
	MDG03	0.9979	1.0008	-	1.0003	1.0000
	MDG04	0.9962	1.0009	0.9943	-	0.9996
	MDG05	0.9965	1.0007	0.9947	1.0001	-

**Table 3:** PSNR, SSIM, UIQ, NCC, NAE, IF, AD, MD, and SC values for greyscale images used in the experiments reported.

Measurement	Cover Image	Secret Image				
		MDG06	MDG07	MDG08	MDG09	MDG10
PSNR	MDG06	-	44.0579	44.0589	44.0722	44.5901
	MDG07	44.3288	-	44.0589	44.0744	44.5852
	MDG08	44.3354	44.0870	-	44.0485	44.5606
	MDG09	44.2275	44.0000	44.0360	-	44.4522
	MDG10	44.3232	44.0541	44.0407	44.0439	-
SSIM	MDG06	-	0.9398	0.9412	0.9400	0.9449
	MDG07	0.9762	-	0.9759	0.9754	0.9774
	MDG08	0.9535	0.9520	-	0.9507	0.9544
	MDG09	0.9351	0.9342	0.9356	-	0.9361
	MDG10	0.9295	0.9269	0.9280	0.9259	-
UIQ	MDG06	-	0.9386	0.9400	0.9387	0.9436
	MDG07	0.9749	-	0.9746	0.9742	0.9759
	MDG08	0.9516	0.9501	-	0.9490	0.9524
	MDG09	0.9342	0.9333	0.9345	-	0.9352
	MDG10	0.9283	0.9257	0.9267	0.9247	-
NCC	MDG06	-	0.9988	0.9978	0.9999	0.9999
	MDG07	0.9994	-	0.9972	0.9999	0.9999
	MDG08	0.9996	0.9984	-	0.9999	0.9999
	MDG09	0.9998	0.9989	0.9982	-	0.9999
	MDG10	0.9998	0.9987	0.9978	0.9999	-
NAE	MDG06	-	0.0109	0.0109	0.0108	0.0102
	MDG07	0.0151	-	0.0155	0.0155	0.0147
	MDG08	0.0160	0.0165	-	0.0166	0.0156
	MDG09	0.0078	0.0080	0.0079	-	0.0077
	MDG10	0.0094	0.0097	0.0097	0.0097	-
IF	MDG06	-	0.9998	0.9998	0.9998	0.9999
	MDG07	0.9998	-	0.9998	0.9998	0.9998
	MDG08	0.9998	0.9997	-	0.9997	0.9998
	MDG09	0.9999	0.9999	0.9999	-	0.9999
	MDG10	0.9999	0.9999	0.9999	0.9999	-
AD	MDG06	-	0.1738	0.3014	−0.0558	−0.0623
	MDG07	0.0270	-	0.3116	−0.0456	−0.0521
	MDG08	-0.0350	0.1220	-	−0.1076	−0.1141
	MDG09	-0.0837	0.0733	0.2009	-	−0.1629
	MDG10	0.0272	0.1842	0.3118	−0.0454	-
MD	MDG06	-	3	3	3	3
	MDG07	3	-	3	3	3
	MDG08	3	3	-	3	3
	MDG09	3	3	3	-	3
	MDG10	3	3	3	3	-
SC	MDG06	-	1.0023	1.0042	0.9990	0.9989
	MDG07	1.0009	-	1.0053	0.9995	0.9996
	MDG08	1.0006	1.0030	-	0.9993	0.9993
	MDG09	1.0003	1.0020	1.0035	-	0.9994
	MDG10	1.0003	1.0025	1.0043	0.9992	-

**Table 4:** Comparison between proposed protocol and similar quantum-based data hiding techniques in terms of embedding capacity and mean values of PSNR, SSIM, and UIQ.

Mechanism	PSNR	SSIM	UIQ	Capacity
Proposed	44.2314	0.9499	0.9487	2-bit/8-bit
[12]	43.5284	0.9698	0.9695	2-bit/8-bit
[5]	44.0267	--	--	2-bit/8-bit
[20]	46.3353	--	--	2-bit/8-bit
